# Supplementary figures and images for: Distinct and Overlapping Functions of Miscanthus sinensis MYB Transcription Factors SCM1 and MYB103 in Lignin Biosynthesis
Source: Int J Mol Sci. 2021 Nov 17;22(22):12395. doi: 10.3390/ijms222212395 (PMC8625797; doi:10.3390/ijms222212395)

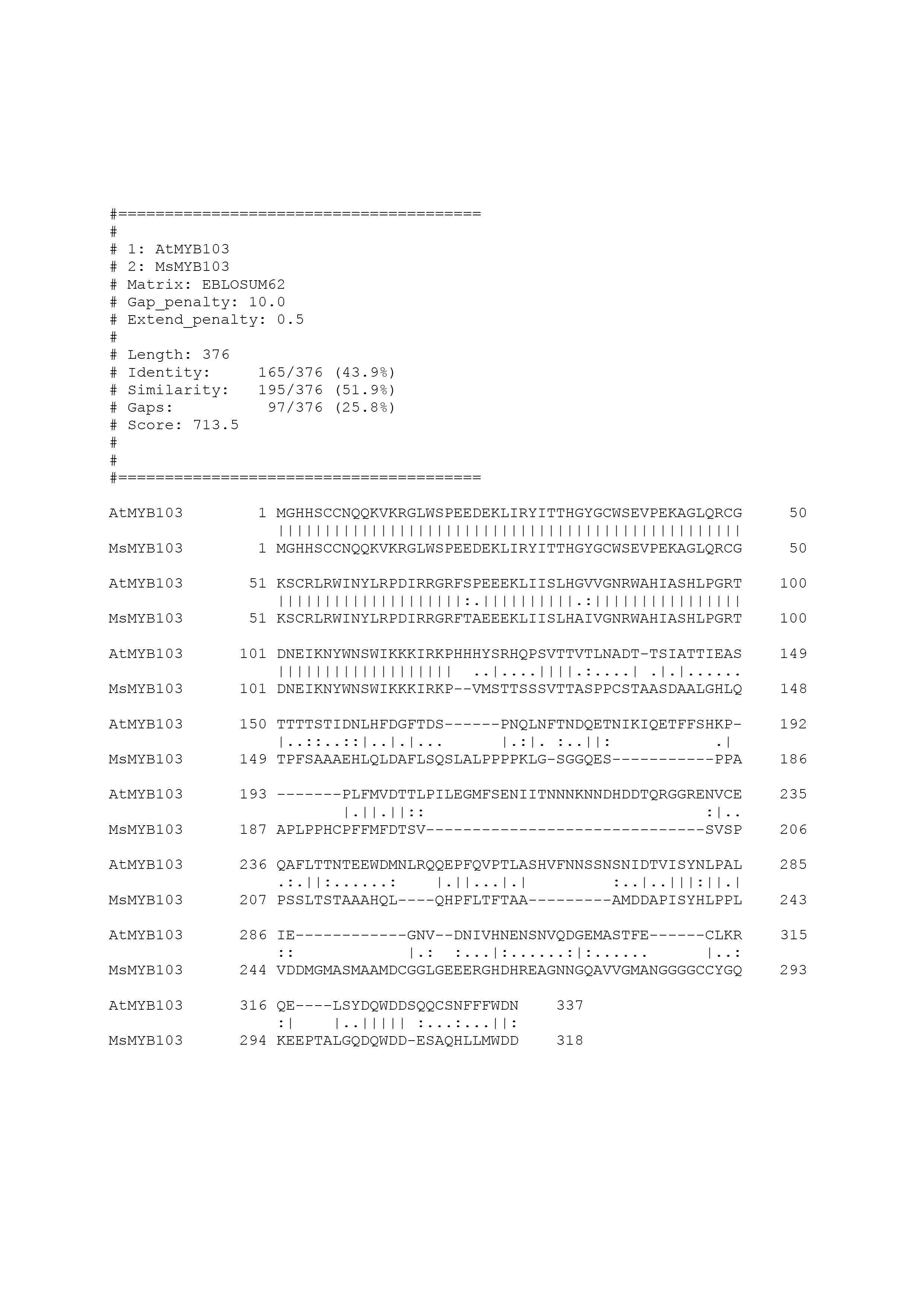

Supplement: Supplementary file 1 [file ijms-22-12395-s001.zip › supplementary materials/Figure S1.tif]

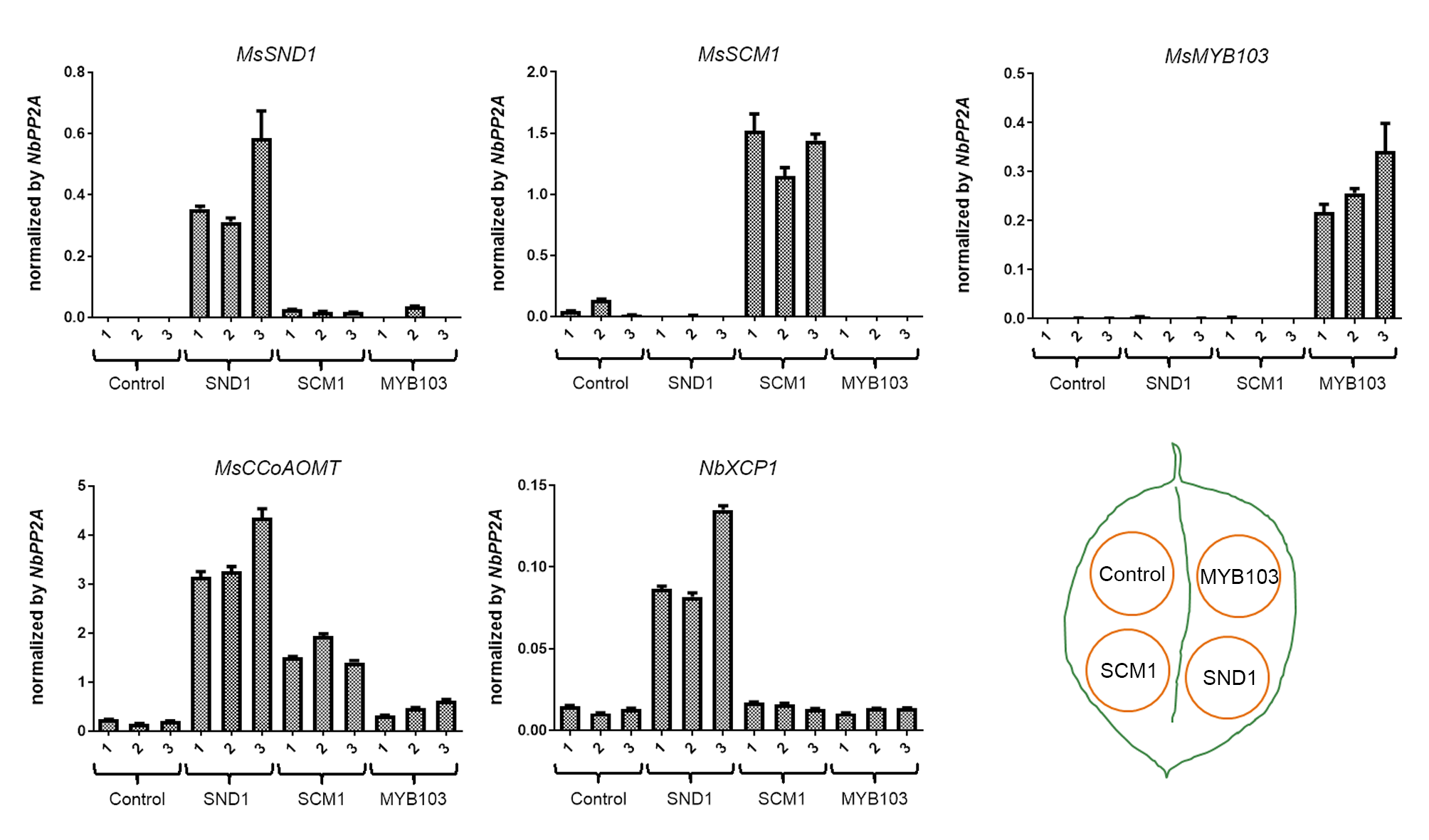

Supplement: Supplementary file 1 [file ijms-22-12395-s001.zip › supplementary materials/Figure S2.tif]
